# “Smart” outsourcing in support of the humanization of entrepreneurship in the artificial intelligence economy

**DOI:** 10.1057/s41599-022-01493-x

**Published:** 2023-01-06

**Authors:** Denis E. Matytsin, Valentin A. Dzedik, Galina A. Markeeva, Saglar B. Boldyreva

**Affiliations:** 1 Volgograd State University, Volgograd, Russia; 2 Moscow State Institute of International Relations (MGIMO University) under the Ministry of Foreign Affairs of the Russian Federation, Moscow, Russia; 3 Kalmyk State University named after B.B. Gorodovikov, Elista, Russia

**Keywords:** Economics, Business and management

## Abstract

The article focuses on the problem of optimizing human resource management with systematic coverage of economic efficiency and corporate social responsibility. The purpose of the article is to study the role of outsourcing in the humanization of entrepreneurship in the AI economy. The potential of outsourcing in the field of entrepreneurship development in the AI economy is justified through econometric modeling using the regression analysis method on the example of companies from the “Global-500” in 2022. Promising directions for the development of “smart” outsourcing in support of the humanization of entrepreneurship in the economy using the method of comparative analysis are determined through the identification of its advantages in comparison with traditional outsourcing for the humanization of entrepreneurship. Using the case study method, successful examples of the use of “smart” outsourcing in various business operations of entrepreneurship are systematized. The key conclusion is that in the AI economy, the humanization of entrepreneurship can be provided with the help of “smart” outsourcing, which is more preferable than traditional outsourcing due to its increased flexibility, rationality, and efficiency. The theoretical significance lies in clarifying the specifics of outsourcing in the AI economy, as well as in justifying the preference for “smart” outsourcing and in providing a scientific methodology for its implementation. The practical significance is connected with the fact that the proposed practical recommendations on the implementation of “smart” outsourcing allow to improve the efficiency of entrepreneurial activity and strengthen the resilience of businesses to economic crises, supporting economic growth through “smart” outsourcing.

## Introduction

Humanization is a new trend in the development of entrepreneurship in support of the implementation of Sustainable Development Goal 8 (SDG 8), which is formulated as “decent work and economic growth” (Moreno, 2022). Corporate social responsibility acts as the main tool for ensuring the humanization of entrepreneurship (Abralava and Javelidze, 2021). The essence of the humanization of entrepreneurship is in creating favorable conditions to develop and fully unlock human potential in the workplace and in labor collectives, in corporate culture and organizational structure (Bluma, 2021; Harris, 2022; Hellström, 2022; Jamali et al., 2022).

The manifestations of the humanization of entrepreneurship are the creation of new (additional) jobs in support of employment (and, accordingly, the fight against unemployment) and occupational employment, the creation of knowledge-intensive and creative (workers relating to innovation activities) jobs, as well as the creation of highly productive and highly paid jobs, as well as providing career opportunities for employees (Ciravegna and Nieri, 2022; Deva, 2022; Greru et al., 2022; Mejia and Aronstein, 2022).

The implementation of SDG8, being the cornerstone of the artificial intelligence economy, hinders the humanization of entrepreneurship within its framework due to the conflicting interests of stakeholders. On the one hand, under the influence of growing digital competition in the conditions of the Fourth Industrial Revolution, enterprises are interested in moving to a higher technological order. This is accompanied by “smart” automation based on artificial intelligence, in which human labor is excluded from a variety of business operations (Akter et al., 2022; Leszkiewicz et al., 2022; Polak, 2021; Washburn, 2019).

This is interpreted positively within the framework of sustainable development, as it supports the practical implementation of Sustainable Development Goal 9(SDG 9), which is formulated as “industrialization, innovation and infrastructure”. It also provides high-tech economic growth, but decreases the need of enterprises for workers and pushes them to reduce staff (Åström et al., 2022; Coltey et al., 2022; Gloor et al., 2022).

On the other hand, workers, society, and the state are interested in fighting unemployment. The artificial preservation of jobs for workers whose functions have been replaced by machines cannot ensure the development of human potential, but it slows down economic growth, and therefore it contradicts SDG 8. The problem becomes even more serious in the conditions of economic crises when economic activity needs to be optimized (Malik et al., 2022).

The requirements for increasing the efficiency of entrepreneurial activity, dictated by the market in conditions of high competition and crisis, force businesses to reduce costs while declining or increasing productivity. In the context of the COVID-19 pandemic and crisis, even socially responsible companies faced the choice between further automation of business operations to maintain production capacities and prolonged business downtime due to the introduced social distancing and lockdown measures (Popkova et al., 2021a, 2021b). The most responsible companies showed ingenuity and transferred employees to remote work with the preservation of wages and social guarantees, even at the expense of the economic efficiency of the business (Popkova et al., 2022).

A promising solution to this problem is outsourcing, which is the transfer of part of business operations to external services with the withdrawal of employees involved in these operations out of the permanent organization’s staff. This makes it possible to save jobs (avoid staff cuts and rising unemployment), but outside the company. The advantages of outsourcing for business are associated with a reduction in the cost of human resource management (HRM), in particular, the costs of recruitment and training of personnel, as well as social (including pension contributions) and tax deductions for employees.

In addition to this, enterprises are able to flexibly change the number and composition of employees depending on the changing needs of human resources. Providing advantages for enterprises, outsourcing does not always comply with the principles of corporate social responsibility and therefore needs special management to humanize entrepreneurship.

The theory and practice of outsourcing have been studied in detail in the existing literature (Inshakova et al., 2022a, 2022b). However, the features of outsourcing in the AI economy are not sufficiently developed and are not clear. This determined the purpose of this article as a study of the role of outsourcing in the humanization of entrepreneurship in the AI economy. The given introduction in this article is followed by a literature review, which includes gap analysis and the formulation of research questions (RQs).

After that, the materials and methodology are described, revealing the order and methodological apparatus of the study. The following are the results of the study, which include: (1) an assessment of the potential of outsourcing in the field of entrepreneurship development in the AI economy and (2) a justification of promising directions for the development of “smart” outsourcing in support of the humanization of entrepreneurship in the AI economy.

Then, in the discussion section, the obtained results are compared with the available literature and the contribution of the article to the field of research is determined. The final part of the article is a conclusion in which the main findings are briefly outlined, the theoretical and practical significance of the authors’ results is identified, and the limitations of this study and the prospects for future scientific research are indicated.

## Literature review

The fundamental basis of the research conducted in this article was formed by the Theory of Human Resource Management (HRM). According to this theory, humanization of entrepreneurship implies responsible management of human resources, which is manifested in the following:

- Preservation and creation of additional jobs to support employment and fight unemployment (Liao et al., 2022; Omidi and Dal Zotto, 2022);
- Optimization and organization of jobs through an increase in their comfort and safety (del-Castillo-Feito et al., 2022; He et al., 2021; Sorribes et al., 2021);
- Provision of opportunities for career building and development of human potential through the creation of knowledge-intensive jobs, with creative activities and innovative activity of employees (Diaz-Carrion et al., 2019; Latan et al., 2022; Rawshdeh et al., 2019; Zhang et al., 2015), as well as the creation of highly productive jobs, which imply the use of the leading technologies and automatization means, in particular, the support of decent jobs for digital personnel (Ramos-González et al., 2022).

The principles and milestones of humanization of entrepreneurship are set in the formulation and description of SDG 8. They are reflected concisely and precisely by the formulation of “decent work”. In the pre-digital era in progressive societies, human resource management implied the manifestation of corporate social responsibility. The need for human resources was high, and therefore the reputation of responsible employers provided enterprises with competitive advantages by attracting and retaining the best personnel in the labor market (Chanda and Goyal, 2020). Responsible HRM manifested itself in increased workplace comfort, strict compliance with labor legislation, as well as the employer’s acceptance of expanded social obligations (Ramos-González et al., 2022).

The transition to the AI economy has radically changed the situation. Although corporate social responsibility is still acutely relevant, the basis of the competitive advantages of modern business is high-tech, achieved through “smart” automation (Inshakova and Anisimov, 2022). At the same time, each case of digital modernization of the enterprise is unique. In some cases, especially in B2C markets, machine technologies displace human resources only in some business operations (Matytsin, 2022a). At the same time, operations involving social communication continue to be carried out with the significant participation of employees of enterprises, which chatbots and “smart” assistants based on artificial intelligence are not yet able to replace (He and Kim, 2021).

In other cases, mainly in B2B markets, fully autonomous “smart” productions are being created, in which the role of human resources is reduced to the maintenance of machine technologies (Sobhani et al., 2021). The third situation involves the most high-tech market segments, where enterprises are interested in continuous digital modernization and high innovation activity (Inshakova et al., 2020). For them, human resources are a key factor in production, as they provide strategic competitive advantages (Matytsin, 2022b). Automation is also beneficial for them, but the loss of valuable personnel and corporate knowledge threatens to lose of market positions (Zhao et al., 2021).

Outsourcing in all considered cases provides a balance of corporate social responsibility, economic efficiency of the business, and its digital competitiveness (Dzedik and Chigirinskaya, 2019). In the first case, business communication with customers can be carried out on the terms of outsourcing (Li et al., 2022). In the second case, technical specialists may be involved in outsourcing to maintain business machine technologies (Iannuzzi and Sacchetto, 2022). In the third case, a business can outsource highly qualified personnel and form innovative work teams from them (Cheung and Kim, 2022).

The problem is that outsourcing weakens the connection between employees and business, so there is a threat of de-evolution of human resources as individualized social capital back to impersonal labor as a factor of production. This makes it difficult to humanize entrepreneurship in the AI economy. In this regard, “smart” outsourcing is proposed as a promising solution to the problem posed in this article, the barrier to the development of which is the lack of formation of the scientific base (Joshi and Raman, 2022; Su et al., 2022).

The conducted literature review revealed a generally high degree of elaboration on the issues of outsourcing in entrepreneurship. However, along with this, two following gaps have been identified. The first gap in the literature is the ambiguity of how much outsourcing is generally in demand in entrepreneurship. In existing publications, individual examples from practice are given and the expediency of using outsourcing in certain sectors of the economy is noted, for example, the electric power sector (Braunert and Figueiredo, 2021), quality control in the automotive industry (Ulewicz, 2018), outsourcing of ax technological processes (Abedi et al., 2014), outsourcing in logistics.

At the same time, it is unclear whether outsourcing is in demand in exceptional cases under certain conditions, it can be widely used. This raises RQ_1_: Can outsourcing be massively applied in entrepreneurship in the AI economy? This article puts forward the H_1_ hypothesis that outsourcing is in demand in entrepreneurship in the AI economy, and its mass application is advisable. Due to its flexibility, outsourcing is universal.

The second gap in the literature is related to the uncertainty of how to outsource in the AI economy. In the available works of Erasmus et al. (2020), Queiroz et al. (2020), Yoon (2021), much attention is paid to the advantages of using “smart” technologies for internal business management. At the same time, the issues of using artificial intelligence in the organization of external business management, in particular, outsourcing, are not sufficiently covered in the literature.

In their works, Agarwal et al. (2022), Furszyfer Del Rio et al. (2020), Popkova et al. (2021) point to the limited availability of “smart” technologies for modern entrepreneurship due to their high cost, the technical complexity of implementation and the need for highly qualified personnel, which not every enterprise has. This causes RQ_2_: How does artificial intelligence improve outsourcing? This article puts forward the H_2_ hypothesis that artificial intelligence allows for “smart” outsourcing, the advantages of which are efficiency and increased flexibility, which make it possible to fully unlock the potential of outsourcing that contributes to the humanization of entrepreneurship.

“Smart” outsourcing refers to the use of “smart” technologies based on artificial intelligence in outsourcing (Annarelli et al., 2022; Reza-Gharehbagh et al., 2022; Zhai et al., 2022). The article aims to fill the identified gaps in the literature, find answers to the research questions (RQs) and test the hypotheses put forward (H_1_ and H_2_). For this purpose, the article systematizes and discusses in detail the practical experience of using outsourcing in entrepreneurship in the AI economy.

## Methods

The article is based on a quantitative and qualitative methodology in accordance with a systematic approach to form the most complete and reliable understanding of the use of outsourcing in entrepreneurship in the AI economy. The research in the article is carried out at the micro-level of the AI economy when studying the practice of entrepreneurship.

To find the answer to RQ_1_ and determine the potential of outsourcing in the field of entrepreneurship development in the AI economy, the regression analysis method is used. With its help, the dependence of revenues (rev) and profit (pft) on the number of employees (ne) is modeled—a simple (one-factor) linear regression is performed. The empirical basis of the study is companies from the “Global-500” rating for 2022 (Fortune, 2022). The research model of this paper is the following system of equations:

1 $$\left\{ \begin{array}{l}{{{\mathrm{rev}}}} = {{{a}}}_{{{{\mathrm{rev}}}}} + {{{b}}}_{{{{\mathrm{rev}}}}} \ast {{{\mathrm{ne}}}}\\ {{{\mathrm{prt}}}} = {{{a}}}_{{{{\mathrm{prt}}}}} + {{{b}}}_{{{{\mathrm{prt}}}}} \ast {{{\mathrm{ne}}}}\end{array} \right.$$

The reliability of the results of econometric modeling is evaluated from the position of coefficients of correlation (*R*-squared) and Significance *F*. The H_1_ hypothesis is considered proven if *b*_rev_ in model (1) turns out to be significantly larger than the *b*_prt_. This will mean that human resources make a much greater contribution to the revenue of companies than to their profits, and therefore outsourcing is beneficial, allowing to retain the number of employees, but withdraw them from the company’s staff.

To find the answer to RQ_2_ and identify promising areas for the development of “smart” outsourcing in support of the humanization of entrepreneurship in the AI economy, the case study method is used. With the help of the chosen method, successful examples of the use of “smart” outsourcing in various business operations of entrepreneurship are systematized. Using the method of comparative analysis, the advantages of “smart” outsourcing compared to traditional outsourcing are identified. The H_2_ hypothesis is recognized as proven if “smart” outsourcing turns out to be preferable and provides advantages for key business operations.

## Results

### Potential of outsourcing in the field of entrepreneurship development in the AI economy

To find the answer to RQ_1_ and determine the potential of outsourcing in the field of entrepreneurship development in the AI economy using the regression analysis method, the dependence of revenues (rev) and profit (pft) on the number of employees (ne) in companies from the “Global-500” for 2022 (Fortune, 2022) was modeled. The following system of equations of simple (one-factor) linear regression is obtained:

2 $$\left\{ \begin{array}{l}{{{\mathrm{rev}}}} = 15,976.76 + 0.27 \ast {{{\mathrm{ne}}}}\\ {{{\mathrm{prt}}}} = 15,976.76 + 0.27 \ast {{{\mathrm{ne}}}}\end{array} \right.$$

The obtained regression Eq. (2) have clarified the research model (1) and indicate that with an increase in the number of employees by 1 person, the revenue of companies from the Global-500 in 2022 increases by $0.27 million, and the profit increases by only $0.022 million. The reliability of the results of econometric modeling is confirmed by regression statistics in Table 1.

**Table 1:** Regression statistics.

Regression statistics	Revenues	Profit
Number of employees (b)	*b*_rev_ = 0.27 (*t*-Stat: 23.22)	*b*_prt_ = 0.02 (t-с Stat: 7.03)
Cons (a)	*a*_rev_ = 15976,76 (*t*-Stat: 8.79)	*a*_prt_ = 2579.66 (*t*-Stat: 6.38)
Obs.	500	500
*R*-squared	0.55199	0.0903
Significance *F*	2.26*10^−81^*	6.8–10^−12^*

Calculated and compiled by the authors.

^*^p < 0.01.

According to Table 1, both regression equations are reliable at a significance level of 0.01. Consequently, human resources contribute much more to the revenue of companies than to their profits. This confirms the H_1_ hypothesis and proves the expediency of the mass use of outsourcing in entrepreneurship in the modern AI-based economy. Thanks to outsourcing, it is possible to preserve the contribution of human resources to the revenue of companies while reducing personnel costs. This will increase the contribution of human resources to the profit of companies.

### Promising directions for the development of “smart” outsourcing in support of the humanization of entrepreneurship in the AI economy

To find the answer to RQ_2_ and identify promising directions for the development of “smart” outsourcing in support of the humanization of entrepreneurship in the economy, the advantages of “smart” outsourcing in comparison with traditional outsourcing for the humanization of entrepreneurship are identified using the comparative analysis method (Table 2).

**Table 2:** Comparative analysis of traditional and “smart” outsourcing in terms of contribution to the humanization of entrepreneurship.

Comparison criteria	Traditional outsourcing	“Smart” outsourcing
Human Resource Management (HRM) Approach	Responsible HRM (corporate social responsibility)	ESG-human resource management
Outsourcing decision-making	Decision-making about outsourcing by a manager taking into account the “human factor”	Intelligent outsourcing decision support
Choosing an outsourcing service provider	Limited market analytics by an outsourcing manager	“Smart”—the most comprehensive analytics of the outsourcing market
Changing the organizational model in outsourcing	A pattern approach to organizational design	“Smart” organizational design
Outsourcing personnel management	Generic personnel management, undermining employee competition	Machine vision: individual approach, “healthy” employee competition
Approach to the introduction of artificial intelligence	Introduction of own artificial intelligence in the enterprise	Outsourcing of artificial intelligence

Developed by the authors.

As shown in Table 2, “smart” outsourcing in theory provides a whole range of advantages for the humanization of entrepreneurship compared to traditional outsourcing. To confirm these advantages in practice, using the case study method, successful examples of the use of “smart” outsourcing in various business operations of entrepreneurship are systematized.

Firstly, the approach to human resource management (HRM) in traditional outsourcing involves responsible HRM with a focus on corporate social responsibility. Within the framework of “smart” outsourcing, ESG management of human resources is carried out, which allows them to be managed not only from the standpoint of corporate social responsibility (*S*) but also from the standpoint of environmental responsibility (*E*—to ensure “green” employment) and from the standpoint of economic efficiency (*G*). For example, IQITO (2022) offers services for “rented IT directors”.

Secondly, within the framework of traditional outsourcing, outsourcing decisions are made by the manager taking into account the “human factor” and the lack of a guarantee of rationality, therefore outsourcing can be implemented where it is unprofitable and impractical. In contrast, “smart” outsourcing provides intelligent support for decision-making about outsourcing. Artificial intelligence is objective and rational, so it recommends outsourcing only where it is really necessary and expedient. For example, the mobile application and the electronic portal “Prodexy” allow determining the optimal conditions for outsourcing using intelligent decision support technologies (IQITO, 2022).

Thirdly, the choice of an outsourcing service provider in traditional outsourcing is carried out through limited market analytics by the outsourcing manager. In contrast, “smart” analytics, which is the most complete analytics of the outsourcing market, guarantees the selection of the optimal provider of outsourcing services according to the entire set of criteria—from their favorable price for the business to the possibilities of humanistic personnel management in outsourcing.

Fourthly, the change of the organizational model in outsourcing traditionally means a pattern approach to organizational design. “Smart” outsourcing, in contrast, provides “smart” organizational design—more flexible and adaptive. For example, the IQITO-360 smart service provides intelligent support for the transfer of employees to remote employment (IQITO, 2022).

Fifthly, outsourcing personnel management traditionally implies generic personnel management with a common system of motivation and stimulation of labor, which undermines employee competition. For example, when working as a team, their contribution is not taken into account. “Smart” outsourcing instead uses machine vision and provides an individual approach to motivating and stimulating the work of each employee by taking into account his individual results. This encourages “healthy” employee competition.

As an example from practice, we can cite the “smart” personnel management service “Sever AI”, which evaluates employees’ resumes using machine learning and automatically invites employees to an interview. The Image Recognition service uses computer vision to monitor and control the individual results of each employee with high accuracy. SFA systems automatically analyze the productivity and other labor characteristics of each employee and also support the competition of personnel based on a system of motivation and stimulation of labor through gamification—the transformation of routine business operations to a game, an interactive competition of employees (Leader Team, 2022).

Sixthly, the approach to the implementation of artificial intelligence traditionally involves the introduction of its artificial intelligence in the enterprise. Due to the high cost, this makes “smart” technologies inaccessible (elite) for business. “Smart” outsourcing implies outsourcing artificial intelligence. This makes it possible to automate business more flexibly, only partially modernizing business operations. At the same time, the majority of employees are retained, and savings in machine technology costs are also achieved.

For example, the company “Smart service” (2022) offers outsourcing of IT infrastructure, including artificial intelligence and “smart” technologies. This ensures the creation of automated workplaces, the organization of remote work of employees, monitoring and ensuring information security, etc. Many large companies in Russia have been using this service—among them retail chains “Lenta”, “Dixie”, “Lamoda”, “Detsky Mir”, “Leonardo”, “Okey”, “Magnet”, as well as “Russian Post”.

The above has formed the evidence base of the H_2_ hypothesis and indicates the preference for “smart” outsourcing due to its advantages for key business operations in support of the humanization of entrepreneurship.

## Discussion

Thus, the article develops and complements the scientific provisions of the Theory of Human Resource Management (HRM). The paper’s contribution to the development of this theory lies in the substantiation of the fact that to implement SDG 8 in the practice of HRM in the AI economy, it is necessary to use “smart” outsourcing, and in the description of its organizational (the foundation on ESG principles) and technological (the use of artificial intelligence (AI) and machine learning (ML)) features and advantages compared to traditional outsourcing. The obtained results are compared with the existing literature in Table 3.

**Table 3:** Comparison of the obtained results with the existing literature.

RQs	Scientific provisions of the available literature	Literature sources	The obtained new scientific results
RQ_1_: Can outsourcing be massively applied in entrepreneurship in the AI economy?	The use of outsourcing in individual cases	Abedi et al. (2014), Braunert and Figueiredo (2021), Cheung and Kim (2022), Iannuzzi and Sacchetto (2022), Joshi and Raman (2022); Su et al. (2022), Li et al. (2022), Ulewicz (2018)	Mass application of outsourcing
RQ_2_: How does artificial intelligence improve outsourcing?	The use of artificial intelligence for the organization of internal business management	Erasmus et al. (2020), Queiroz et al. (2020), Yoon, (2021)	Use of artificial intelligence to organize external business management, including outsourcing
	Relying on own artificial intelligence	Agarwal et al. (2022), Furszyfer Del Rio et al. (2020), Popkova et al. (2021)	Outsourcing of artificial intelligence, including for the purposes of “smart” outsourcing

Source: Developed by the authors.

Unlike Abedi et al. (2014), Braunert and Figueiredo (2021), Cheung and Kim (2022), Iannuzzi and Sacchetto (2022), Joshi and Raman (2022), Su et al. (2022), Li et al. (2022), Ulewicz (2018), the article proved that the use of outsourcing should not be limited to individual cases, and it is advisable to massively use outsourcing to improve the efficiency of human resource management in entrepreneurship. Thus, the key role of outsourcing in the implementation of SDG 8 in the AI economy is justified.

Unlike Erasmus et al. (2020), Queiroz et al. (2020), Yoon (2021), the article proved that the use of artificial intelligence is appropriate not only for the organization of internal business management but also for the organization of external business management, including outsourcing. For this purpose, “smart” outsourcing is recommended, involving reliance on artificial intelligence and “smart” technologies in all business operations related to outsourcing, including decision-making on outsourcing, choosing an outsourcing service provider, changing the organizational model in outsourcing, as well as personnel management in outsourcing.

Unlike Agarwal et al. (2022), Furszyfer Del Rio et al. (2020), Popkova et al. (2021), the article proved that reliance on their own artificial intelligence is not mandatory. Instead, it is possible to outsource artificial intelligence, including for the purposes of “smart” outsourcing, which is becoming massively available and even more effective.

The contribution of the article to the literature consists in rethinking the essence and role of outsourcing in the AI economy. The obtained results showed outsourcing in a new light, as it is presented for the first time not only as a tool but also as an object of automation. The key conclusion of the study is that in the AI economy, the humanization of entrepreneurship can be provided with the help of “smart” outsourcing, which is more preferable than traditional outsourcing due to its increased flexibility, rationality and efficiency.

## Conclusion

So, the article filled the identified gaps in the literature, provided answers to the research questions (RQs) and proved the hypotheses put forward (H_1_ and H_2_). As an answer to RQ_1_, the article proved the H_1_ hypothesis and justified, using the experience of companies from the Global-500 rating in 2022, that outsourcing is in demand in entrepreneurship in the AI economy, and its mass application is advisable. Outsourcing makes it possible to increase the contribution of human resources to profit compared to the same contribution of the permanent company’s staff.

As a response to RQ_2_, the article confirmed the H_2_ hypothesis and proved that artificial intelligence enables “smart” outsourcing, using its advantages such as increased flexibility and efficiency, which makes it possible to fully unlock the potential of outsourcing, thereby contributing to the humanization of entrepreneurship.

The advantages of “smart” outsourcing are revealed in the entire spectrum of outsourcing-related business operations. “Smart” outsourcing provides a more advanced approach to human resource management (HRM)—ESG–human resource management. Intellectual support for decision-making about outsourcing contributes to the humanization of entrepreneurship through a harmonious combination of corporate social responsibility and business economic efficiency.

Both “Smart” analytics, which is the most comprehensive analysis of the outsourcing market, and “smart” organizational design make it possible to mitigate as much as possible the social consequences of outsourcing, including remote employment. Machine vision provides an individual approach to outsourcing personnel management, and also stimulates “healthy” employee competition. A new approach to the introduction of artificial intelligence, based on its outsourcing, makes “smart” outsourcing massively accessible, including for small and medium-sized businesses and for local businesses.

Thus, the article solves the problem and proves that “smart” outsourcing enables the maintenance of existing and establish new connections between employees and businesses. “Smart” outsourcing adapts not only to the needs of the business but also to the characteristics of each employee. It can take the form of remote employment, which in the conditions of the COVID-19 pandemic and crisis has been successfully used to preserve the social capital of business and support employment.

“Smart” outsourcing supports the further evolution of human resources, which are becoming individual social capital and a subject of competition for enterprises. This fundamentally changes the situation in the labor market—the desire of employees to maintain current employment conditions is replaced by the competition of enterprises for the preservation of personnel provided to them on the terms of outsourcing and professional teams formed from them. Thanks to this, corporate knowledge and human resources become even more valuable for business. “Smart” outsourcing makes it possible to transfer corporate social responsibility from the category of a limiting factor to the category of a source of enterprise efficiency. This opens up new prospects for the humanization of entrepreneurship.

The key conclusion of this paper for the theory and practice of humanization of entrepreneurship is that under the conditions of the AI economy, traditional outsourcing is to be replaced with “smart” outsourcing. For the widest, quickest, and most effective humanization of entrepreneurship, “smart” outsourcing should supplement the management of own human (and intellectual) resources at a company and fully replace traditional outsourcing of these resources. This will allow for the successful and full implementation of SDG 8 in the Decade of Action: ensure socially responsible management of human resources of companies and, at the same time, strengthen their digital competitive advantages and support high-tech economic growth.

The theoretical significance lies in clarifying the specifics of outsourcing in the AI economy, as well as in justifying the preference for “smart” outsourcing and in providing a scientific methodology for its implementation. The practical significance of the results obtained in the article is due to the fact that the proposed practical recommendations for the implementation of “smart” outsourcing make it possible to improve the efficiency of entrepreneurial activity and strengthen the resilience of businesses to economic crises, support economic growth through “smart” outsourcing.

The social significance of the article is explained by the fact that it formed a scientific vision and outlined the prospects for a harmonious and balanced practical implementation of SDG 8 and SDG 9 by unlocking the potential of humanization of entrepreneurship in the economy of artificial intelligence based on “smart” outsourcing.

The limitation of the obtained results is the social aspect of the study of “smart” outsourcing from the standpoint of the humanization of entrepreneurship in the AI economy. The chosen perspective allowed us to highlight organizational issues and social benefits of “smart” outsourcing in terms of corporate social responsibility within the framework of this study. At the same time, technical issues remained outside the scope of the study—they will be revealed by future scientific research.

## Data Availability

The article is based on the official Global-500 statistics for 2022 (Fortune, 2022). For rechecking and reproducibility of the results obtained, an array of data is attached to this article in a Microsoft Excel table (Data Table) available for automated data analytics. Link to the data source: Fortune (2022). Global-500, 2022. https://fortune.com/fortune500/ (data accessed: 19.08.2022).
